# Acute lower respiratory infections in ≥5 year -old hospitalized patients in Cambodia, a low-income tropical country: clinical characteristics and pathogenic etiology

**DOI:** 10.1186/1471-2334-13-97

**Published:** 2013-02-22

**Authors:** Sirenda Vong, Bertrand Guillard, Laurence Borand, Blandine Rammaert, Sophie Goyet, Vantha Te, Patrich Lorn Try, Sopheak Hem, Sareth Rith, Sowath Ly, Philippe Cavailler, Charles Mayaud, Philippe Buchy

**Affiliations:** 1 Institut Pasteur in Cambodia, Réseau International des Instituts Pasteur, Phnom Penh, Cambodia; 2 Takeo provincial hospital, Takeo, Cambodia; 3 Kampong Cham provincial hospital, Kampong Cham, Cambodia; 4 Hôpital Tenon, Assistance Publique - Hôpitaux de Paris, Paris, France

**Keywords:** Cambodia, Acute lower respiratory infection, Tropics, Hospitalized patients, Viruses, Bacteria, Adults, Older children

## Abstract

**Background:**

Few data exist on viral and bacterial etiology of acute lower respiratory infections (ALRI) in ≥5 year –old persons in the tropics.

**Methods:**

We conducted active surveillance of community-acquired ALRI in two hospitals in Cambodia, a low-income tropical country. Patients were tested for acid-fast bacilli (AFB) by direct sputum examination, other bacteria by blood and/or sputum cultures, and respiratory viruses using molecular techniques on nasopharyngeal/throat swabs. Pulmonologists reviewed clinical/laboratory data and interpreted chest X-rays (CXR) to confirm ALRI*.*

**Results:**

Between April 2007 - December 2009, 1,904 patients aged ≥5 years were admitted with acute pneumonia (50.4%), lung sequelae-associated ALRI (24.3%), isolated pleural effusions (8.9%) or normal CXR-related ALRI (17.1%); 61 (3.2%) died during hospitalization. The two former diagnoses were predominantly due to bacterial etiologies while viral detection was more frequent in the two latter diagnoses. AFB-positive accounted for 25.6% of acute pneumonia. Of the positive cultures (16.8%), abscess-prone Gram-negative bacteria (39.6%) and *Haemophilus influenzae* (38.0%) were most frequent, followed by *Streptococcus pneumoniae* (17.7%). Of the identified viruses, the three most common viruses included rhinoviruses (49.5%), respiratory syncytial virus (17.7%) and influenza viruses (12.1%) regardless of the diagnostic groups. Wheezing was associated with viral identification (31.9% vs. 13.8%, p < 0.001) independent of age and time-to-admission.

**Conclusions:**

High frequency of *H. influenzae* and *S. pneumoniae* infections support the need for introduction of the respective vaccines in the national immunization program. Tuberculosis was frequent in patients with acute pneumonia, requiring further investigation. The relationship between respiratory viruses and wheezing merits further studies.

## Background

Pneumonia and other acute lower respiratory infections (ALRI) are the third leading cause of death worldwide and the first in low-income countries [1]. The vast majority of ALRI deaths are due to pneumonia [1] and nearly all severe ALRI episodes occur in children under 5 years, the elderly and immuno-compromised individuals (e.g. HIV-infected). Globally, approximately 4.2 million deaths caused by ALRI are estimated to occur annually across all age groups [2]. ALRI is a disease of all ages in low-income countries; it is one of the most common diagnoses upon admission [3].

Despite this burden and the development of improved microbiological molecular methods, only few recent studies with well characterized ALRI, clinical diagnoses in which bacterial and viral testing have been additionally performed have been published [4-7]. In general, the etiology of ALRI, and pneumonia in particular, remains difficult to establish [8,9] using either insensitive or non-specific indirect methods such as blood culture, diagnostic serology and pathogenic detection of the upper airway [10]. In many low-income countries, most hospital laboratories do not possess the capacity for microbiologic diagnostic testing and pneumonia is still defined clinically using radiographs which are not always available in many health facilities. Taken together, the epidemiology of ALRI remains poorly defined across the developing world.

We collaborated with two Cambodian provincial hospitals to determine the etiology of community-acquired ALRI. This report summarizes the microbiological, radiological and clinical findings with a focus on patients aged 5 years and older.

## Methods

### Study site and study population

Cambodia (estimated 2008 population ~14.6 Million) [11] is a tropical country in Southeast Asia. The country has very poor health indicators with life expectancy at birth of 59 years and an infant mortality of 90.9/1,000 live births [11]. The study hospitals were located in Takeo province (estimated population 0.9 Million) and Kampong Cham province (estimated pop. 1.7 Million) [11], both within ~150 km of Phnom Penh, the capital. Like most Cambodian public hospitals, the selected study hospitals were ill-equipped with poor laboratory capacity for microbial identification, possessed no CT scanners, and had inadequate intensive care units (supplemental oxygen available but no mechanical ventilation).

### Study design

We enrolled patients aged ≥5 years hospitalized with a clinical syndrome compatible with ALRI, which was defined as (1) symptom onset ≤14 days AND (2) a fever of ≥38°C or a history of febrile episodes during the last 3 days AND (3) cough, AND at least one of the following respiratory symptom or signs: dyspnea, chest pain, or crackles on lung auscultation. Patients with known tuberculosis (TB), HIV or active cancer were excluded. Decision whether to hospitalize was left to the clinicians’ judgment. Demographic information, basic clinical features, past medical history and use of antibiotics prior to admission were collected on a standardized case report form (CRF). Recorded laboratory and microbiology findings, antibiotic treatment and outcome status upon discharge were also collected. Independent expert pulmonologists unaware of the microbiologic diagnosis retrospectively interpreted each CXR. They subsequently assigned the final diagnosis of the various types of ALRI in this study based on a combination of the CXR interpretation and information from the CRF. Written informed consent was obtained from patients aged 18 years and older and the parents or guardians of children. The study was approved by the Cambodian National Ethical Committee for Medical Research.

### Specimen collection

Blood cultures, non-induced sputum samples, and throat and nasopharyngeal swabs (Tubed Sterile Dryswab® cotton-tip) were to be all collected from each participant within 48 hours after hospital presentation for direct examination, culture and molecular diagnostic testing. Cultures and molecular techniques were performed at the Institut Pasteur - Cambodia (IPC) in Phnom Penh. Blood Hemoline Diphasic Performance culture bottles (BioMérieux) were stored in an incubator at 37°C. The incubator was provided by the surveillance project and was transported daily along with sputum which had been collected in the preceding 12 hours. Swabs were immediately placed in a viral transport medium after collection, which was containers consisting of frozen liquid nitrogen. As part of the National Tuberculosis Control Program, direct sputum examination for acid-fast bacilli (AFB) testing was performed on admission and repeated during the subsequent two days in the hospital laboratories. White cell counts and blood chemistries were also performed in the hospital laboratories. The provider-initiated testing and counseling policy was initiated in 2007 in Cambodia. This policy integrates routine HIV testing for patients with sexually transmitted infections or TB, pregnant women, and hospitalized patients suspected of being infected with HIV (per the National HIV/AIDS program; http://www.nchads.org).

### Microbiology testing

All nasopharyngeal and throat samples were tested using 5 validated multiplex reverse transcription polymerase chain reaction (RT-PCR)/PCR for the presence of 18 viral respiratory pathogens including human metapneumovirus, respiratory syncytial virus (RSV), human bocavirus, Influenza A and B viruses, coronaviruses (OC43, 229E, HKU-1, NL63, SARS), parainfluenza viruses 1–4, adenoviruses, rhinovirus and enteroviruses, as previously described elsewhere [12,13]. Sputum specimens were stained using the May-Grünwald-Giemsa method*.* Only good-quality specimens were submitted for bacterial cultures (defined as >25 polynuclear leukocytes and <25 squamous epithelial cells per low-power field) [14]. Sputum specimens were inoculated on colistin-nalidixic acid agar (CNA), cystine-lactose-electrolyte-deficient agar (CLED), chocolate polyvitex agar with bacitracin, and Ashdown agar. Bacterial isolates were identified by API gallery (BioMérieux).

### Case definitions

A bacteriological diagnosis was confirmed when a pathogen was isolated from an uncontaminated blood culture or sputum specimen obtained on admission. A significant growth of bacteria was defined as greater than 10^7^ organisms per ml of original sputum. TB infection was defined by a positive acid-fast-bacilli (AFB) smear, as 97% of them were linked to a positive culture for *Mycobacterium tuberculosis* in Cambodian hospitalized patients (IPC’s unpublished data). Three external expert pulmonologists concurred that a severe case would be defined by the presence of at least 2 of the following criteria: systolic blood pressure <90 mmHg, heart rate ≥120 beats per minute, respiratory rate ≥30/min, oxygen saturation <90% measured by pulse oximeter, body temperature <35°C or ≥40°C; international severity indices (e.g. PSI or CURB65) for pneumonia could not be applied in absence of ICUs or blood urea testing. Pneumonia was defined by the radiologic images of lobar consolidation, nodules, alveolar or interstitial infiltrates; a necrotizing pneumonia meant presence of cavitations. Preexisting pulmonary lesions may alter or disguise the appearance of a pneumonic infiltrate. Consequently we created a separate diagnostic group, ALRI with lung sequelae which was defined as radiological images of cavitary sequelae, partial collapse or bronchiectasis. The ALRI with the normal parenchyma group consisted of patients presenting with symptoms of acute bronchitis and normal lung radiographs.

### Data analysis

We included reliable clinical signs and symptoms, major laboratory findings, reports of medical history, antibiotic use during and preceding hospitalization, and the final diagnosis provided by the pulmonology experts in our analysis. Statistical differences between various groups were detected using either Chi-squared or adjusted Chi-squared test, Chi-squared for trend test, and non-parametric (Kruskal-Wallis) test as appropriate. Proportions, odds ratios (OR), adjusted ORs (aOR) and p values were calculated using STATA version 9.0 (Stata corp., College Station, TX, USA). We used the Mantel-Haenszel test for bivariate analysis and the Wald test in the logistic regression model retaining variables with a p value of ≤ 0.1.

## Results

### Patient characteristics

**Figure 1:**
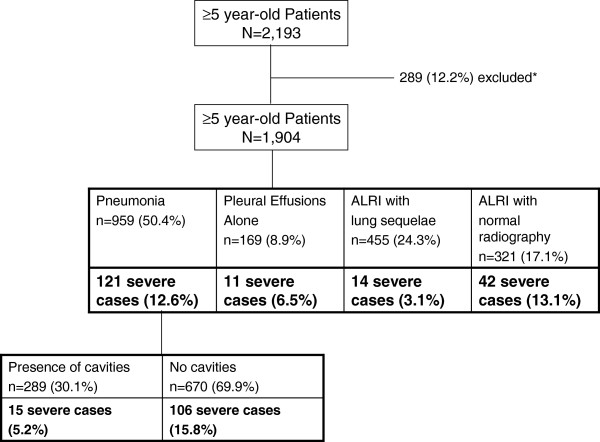
**Various clinical diagnostic groups among patients aged ≥5 years and hospitalized with acute lower respiratory infection, Kampong Cham and Takeo provinces, Cambodia, April 2007 – December 2009.** *Reasons for exclusion: absence of chest X-ray (11.4%); patients ascertained as having extra-pulmonary diseases (9.9%) or upper-respiratory infections (0.5%); 8 patients who also had extra-pulmonary site of infection and those who reported a history of hospitalization within the 2 preceding weeks.

During April 2007 – December 2009, of 2,193 hospitalized patients aged ≥5 years who met the eligibility criteria, 1,904 patients were confirmed to have a diagnosis of ALRI and their data were analyzed (Figure 1). The demographic characteristics of the patients and their diagnoses are presented in Table 1.

**Table 1:** Characteristics of 1904 patients aged ≥5 years hospitalized with community-acquired acute lower respiratory infection from April 2007 to December 2009, Kampong Cham and Takeo provinces, Cambodia

		**Pneumonia [1]**			**Pleural Effusions alone [2]**	**ALRI with sequelae [3]**	**ALRI with normal CXR [4]**	**P values from comparisons between group [1], [2], [3] and [4] by Chi-squared or adjusted Chi-squared test**				
	**Grand total**	**Total**	**Cavitations [1a]**	**No cavitations[1b]**	**n (%)**	**n (%)**	**n (%)**	**[1a] vs. [1b]**	**[1] vs.[2]**	**[1] vs.[3]**	**[1] vs. [4]**	**[3] vs. [4]**
	**1904 (%)**	**959 (50.4)**	**289 (30.1)**	**670 (69.9)**	**169 (8.9)**	**455 (24.3)**	**321 (17.1)**					
Median age in years (IQR*)	53 (38–65)	51	50	52 (35–65)	52 (40–66)	60 (51–70)	44 (17–59)	NS	NS	**<0.001**	**<0.001**	**<0.001**
**Mean age in years**	50.0	48.6	49.3	48.3	51.0	59.0	40.5	NS	NS	**<0.001**	**<0.001**	**<0.001**
**Age groups in years**												
5-14	154 (8.1%)	82 (8.6)	0 (0.0)	82 (12.2)	3 (1.8)	1 (0.2)	68 (21.2)	**<0.001**	**<0.001**	**<0.001**	**<0.001**	**<0.001**
15 - 49	659 (34.6%)	368 (38.4)	144 (49.8)	224 (33.4)	72 (42.6)	97 (21.3)	122 (38.0)	**<0.001**	NS	**<0.001**	**NS**	**<0.001**
≥50	1091 (57.3)	509 (53.1)	145 (50.2)	364 (54.3)	94 (55.6)	357 (78.5)	131 (40.8)	0.249	NS	**<0.001**	**<0.001**	**<0.001**
**Gender, male (%)**	941 (49.4)	502 (52.3)	157 (54.3)	345 (51.5)	90 (53.3)	205 (45.1)	144 (44.9)	NS	NS	**0.011**	**0.026**	NS
**Signs and symptoms on admission**												
Wheezing	261 (13.9)	105 (11.1)	13 (4.5)	92 (14.0)	3 (1.8)	50 (11.0)	103 (32.8)	**<0.001**	**<0.001**	NS	**<0.001**	**<0.001**
Dry cough	519 (27.3)	229 (23.9)	39 (13.5)	190 (28.4)	111 (65.7)	66 (14.5)	113 (35.2)	**<0.001**	**<0.001**	**<0.001**	**<0.001**	**<0.001**
**Time interval between presentation and symptoms onset in days (# of missing values = 112)**												
Median (IQR)	6 (4–9)	6 (4–9)	7 (4–10)	5 (4–9)	6 (4–10)	6 (4–10)	4 (4–8)	**<0.001**	NS	**0.032**	**0.004**	**<0.001**
Mean	6.3	6.3	7.0	5.9	6. 6	6.7	5.6	**<0.002**	NS	0.072	**0.003**	**<0.001**
**Duration of hospital stay in days**												
Median (IQR)	7 (4–11)	7 (4–10)	6 (4–10)	7 (4–10)	5 (3–8)	**9 (6–14)**	7 (4–10)	NS	**<0.001**	**<0.001**	NS	**<0.001**
Mean	8.4	7.9	7.8	8	6.5	10.7	7.7	NS	**<0.001**	**<0.001**	NS	**<0.001**
**Reports of medicine use prior to admission**												
No antibiotic used	932 (48.9)	455 (47.4)	116 (40.1)	339 (50.6)	103 (60.9)	211 (46.4)	163 (50.8)	**0.004**	0.002	NS	NS	NS
Intake of unknown origin	776 (40.8)	405 (42.2)	143 (49.5)	262 (39.1)	43 (25.4)	211 (46.4)	117 (36.4)	**0.004**	**<0.001**	NS	0.078	**0.013**
Antibiotic used	196 (10.3)	99 (10.3)	30 (10.4)	69 (10.3)	23 (13.6)	33 (7.3)	41 (12.8)	NS	NS	0.087	NS	**0.014**
**Patient outcomes**												
Deceased	61 (3.2)	43 (4.5)	6 (2.1)	37 (5.5)	5 (3.0)	11 (2.4)	2 (0.6)	**0.021**	NS	0.076	0.003	NS
Cured or improved	1264 (66.4)	572 (59.6)	117 (40.5)	455 (67.9)	79 (46.7)	321 (70.5)	292 (91.0)	**<0.001**	**0.002**	**<0.001**	**<0.001**	**<0.001**
Transferred	482 (25.3)	297 (31.0)	154 (53.3)	143 (21.3)	82 (48.5)	89 (19.6)	14 (4.4)	**<0.001**	**<0.001**	**<0.001**	**<0.001**	**<0.001**
Escaped	97 (5.1)	47 (4.9)	12 (4.2)	35 (5.2)	3 (1.8)	34 (7.5)	13 (4.0)	NS	NS	0.066	NS	**0.031**
**Reported co-morbid conditions**												
Cardiovascular disorder	213 (11.2)	129 (13.5)	9 (3.1)	120 (17.9)	17 (10.1)	28 (6.2)	39 (12.1)	**<0.001**	NS	**<0.001**	NS	0.003
Diabetes mellitus	195 (10.2)	111 (11.6)	32 (11.1)	79 (11.8)	22 (13.0)	40 (8.8)	22 (6.9)	NS	NS	NS	**0.023**	NS
Renal disorder	136 (7.1)	80 (8.3)	25 (8.7)	55 (8.2)	8 (4.7)	33 (7.3)	15 (4.7)	NS	NS	NS	**0.044**	NS
Liver disorder	16 (0.8)	10 (1.0)	3 (1.0)	7 (1.0)	3 (1.8)	1 (0.2)	2 (0.6)	NS	NS	NS	NS	NFS
**Severe cases**	188 (9.9)	121 (12.6)	15 (5.2)	106 (15.6)	11 (6.5)	14 (3.1)	42 (13.1)	**<0.001**	**0.032**	**<0.001**	NS	**<0.001**
Laboratory results**												
Leukocytosis (missing values = 12)	663 (34.8)	399 (41.9)	120 (41.7)	279 (42.2)	42(25.3)	133 (29.4)	89 (27.8)	NS	**<0.001**	**<0.001**	**<0.001**	NS
Elevated neutrophils count (missing values = 65)	629 (33.0)	378 (40.9)	114 (40.6)	264 (41.1)	40 (24.4)	127 (28.9)	84 (27.0)	NS	**<0.001**	**<0.001**	**<0.001**	NS
Lymphocytosis (missing values = 314)	99 (5.2)	46 (5.5)	7 (3.0)	39 (6.4)	8 (5.0)	22 (7.7)	23 (7.5)	**0.004**	NS	NS	NS	NS
**Patients tested by microbiology method (excluding contaminated samples or cultures)**												
Blood culture	1695 (89.0)	862 (89.9)	264 (91.3)	598 (89.3)	149 (88.2)	412 (90.5)	272 (84.7)	NS	NS	NS	**0.015**	**0.012**
Sputum culture	888 (46.6)	464 (48.4)	185 (64.0)	279 (41.6)	37 (21.9)	270 (59.3)	117 (36.4)	**<0.001**	**<0.001**	**<0.001**	**0.002**	**<0.001**
Blood or sputum culture	1789 (94.0)	905 (94.4)	280 (96.9)	625 (93.3)	152 (89.9)	437 (96.0)	295 (91.9)	NS	NS	NS	NS	**0.016**
Sputum smear for acid-fast bacilli (AFB)	1682 (88.3)	850 (88.6)	280 (96.9)	570 (85.1)	140 (82.8)	434 (95.4)	258 (80.4)	NS	NS	**<0.001**	**0.001**	**<0.001**
Nasopharyngeal/throat swabs by PCR (a)	1855 (97.4)	935 (97.5)	281 (97.2)	654 (97.6)	164 (97.0)	442 (97.1)	314 (97.8)	NS	NS	NS	NS	NS
Samples collected for bacterial & AFB etiologies (b)	1796 (94.3)	911 (95.0)	283 (97.9)	628 (93.7)	152 (89.9)	438 (96.3)	295 (91.9)	NS	NS	NS	NS	**0.008**
Samples collected for bacterial & viral etiologies (c)	1747 (91.8)	887 (92.5)	275 (95.2)	612 (91.3)	147 (87.0)	425 (93.4)	288 (89.7)	NS	NS	NS	NS	0.064
**Bacterial or viral yield ‡**												
Positive blood culture	33 (2.0)	27 (3.1)	5 (1.9)	22 (3.7)	4 (2.7)	2 (0.5)	0 (0.0)	NS	NS	**0.007**	**0.007**	NS
Positive sputum culture	272 (30.6)	138 (29.7)	44 (23.8)	94 (33.7)	8 (21.6)	95 (35.2)	31 (26.5)	**0.029**	NS	NS	NS	NS
Positive blood or sputum culture (d)	300 (16.8)	161 (17.8)	47 (16.8)	114 (18.2)	12 (7.9)	96 (22.0)	31 (10.5)	NS	**0.003**	0.079	**0.007**	**<0.001**
Positive AFB smear (e)	264 (15.7)	218 (25.7)	153 (54.6)	65 (11.4)	2 (1.4)	42 (9.7)	2 (0.8)	**<0.001**	**<0.001**	**<0.001**	**<0.001**	**<0.001**
Positive PCR for viruses (f)	305 (16.4)	138 (14.8)	22 (7.8)	116 (17.7)	17 (10.4)	68 (15.4)	82 (26.1)	**<0.001**	NS	NS	**<0.001**	**0.004**
**Bacterial etiologies†**												
*H. influenzae*	114 (38.0)	52 (32.3)	13 (27.7)	39 (34.2)	0 (0.0)	46 (47.9)	16 (51.6)	NS	**0.005**	**0.002**	NS	**0.022**
*- In sputum*^1^	114 (41.9)	52 (37.7)	13 (29.5)	39 (41.5)	0 (0.0)	46 (48.4)	16 (51.6)					
*- In blood*^2^	1 (3.0)	1 (3.7)	1 (20.0)	0 (0.0)	0 (0.0)	0 (0.0)	0 (0.0)					
*S. pneumoniae*	53 (17.7)	22 (13.7)	6 (12.8)	16 (14.0)	1 (8.3)	23 (24.0)	7 (22.6)	NS	NS	**0.009**	NS	NS
*- In sputum*^1^	51 (18.8)	20 (14.5)	6 (13.6)	14 (14.9)	1 (12.5)	23 (24.2)	7 (22.6)					
*- In blood*^2^	2 (6.0)	2 (7.4)	0 (0.0)	2 (9.1)	0 (0.0)	0 (0.0)	0 (0.0)					
*B. pseudomallei*	28 (9.3)	25 (15.5)	10 (21.3)	15 (13.2)	1 (8.3)	1 (1.0)	1 (3.2)	NS	NS	**<0.001**	**0.020**	NS
*- In sputum*^1^	16 (5.9)	14 (10.1)	7 (15.9)	7 (7.4)	1 (12.5)	0 (0.0)	1 (3.2)					
*- In blood*^2^	15 (45.5)	14 (51.9)	4 (9.0)	10 (10.6)	0 (0.0)	1 (1.1)	0 (0.0)					
*K. pneumoniae*	43 (14.3)	28 (17.4)	10 (21.3)	18 (15.8)	2 (16.7)	8 (8.3)	5 (16.1)	NS	NS	NS	NS	NS
*- In sputum*^1^	42 (15.4)	27 (19.6)	10 (22.7)	17 (18.1)	2 (25.0)	8 (8.4)	5 (16.1)					
*- In blood*^2^	1 (3.0)	1 (3.7)	0 (0.0)	1 (4.6)	0 (0.0)	0 (0.0)	0 (0.0)					
*P. aeruginosa*	37 (12.3)	19 (11.8)	6 (12.8)	13 (11.4)	1 (8.3)	15 (15.6)	2 (6.5)	NS	NS	NS	NS	**0.037**
*S. aureus*	11 (3.7)	5 (3.1)	2 (4.3)	3 (2.6)	1 (8.3)	4 (4.2)	1 (3.2)	NS	NS	NS	NS	NS
Other bacteria	39 (13.0)	22 (13.7)	4 (8.5)	18 (15.8)	7 (58.3)	9 (9.4)	1 (3.2)	NS	NS	NS	**0.040**	NS
AFB-positive***	264 (15.7)	218 (25.6)	153 (54.6)	65 (11.4)	2 (1.4)	42 (9.7)	2 (0.8)	**<0.001**	**<0.001**	**<0.001**	**<0.001**	**<0.001**
**Positive PCR results ^**												
Rhinovirus	151 (49.5)	68 (49.3)	10 (45.5)	58 (50.0)	8 (47.1)	30 (44.1)	45 (54.9)	**0.007**	NS	NS	**<0.001**	**0.001**
Respiratory syncytial virus	54 (17.7)	23 (16.7)	5 (23.7)	18 (15.5)	5 (29.4)	11 (16.2)	15 (18.3)	NS	NS	NS	0.064	NS
Influenza viruses	37 (12.1)	16 (11.6)	3 (13.6)	13 (11.2)	0 (0.0)	11 (16.2)	10 (12.2)	NS	NS	NS	NS	NS
Coronaviruses	31 (10.2)	18 (13.0)	2 (9.9)	16 (13.8)	1 (5.9)	11 (16.2)	1 (1.2)	NS	NS	NS	0.080	**0.001**
Human metapneumovirus	14 (4.6)	7 (5.1)	0 (0.0)	7 (6.0)	2 (11.8)	2 (2.9)	3 (3.7)	NS	NS	NS	NS	NS
Other viruses	20 (6.6)	6 (4.3)	2 (9.1)	4 (3.5)	2 (11.8)	5 (7.4)	7 (8.5)	NS	NS	NS	NS	NS
**Mixed infections**												
>1 pathogen (AFB, bact. or virus) (% = n*100/d)	63 (21.0)	35 (21.7)	16 (34.0)	19 (16.7)	2 (16.7)	22 (22.9)	4 (12.9)	NS	NS	NS	NS	NS
>1 bacterium identified (% = n*100/d)	24 (8.0)	12 (7.5)	4 (8.5)	8 (7.0)	0 (0.0)	10 (10.4)	2 (6.5)	NS	NS	NS	NS	NS
AFB and ≥1 bacterium identified (% = n*100/d)	30 (10.0)	24 (14.9)	16 (34.0)	8 (7.0)	0 (0.0)	6 (6.3)	0 (0.0)	NS	NS	NS	NS	NS
AFB & virus (% = n*100/f)	26 (8.5)	18 (13.0)	14 (63.6)	4 (3.5)	0 (0.0)	8 (11.8)	0 (0.0)	**0.005**	NS	NS	NS	NS
>1 virus (% = n*100/f)	9 (3.0)	3 (2.2)	1 (4.5)	2 (1.7)	2 (1.7)	2 (2.9)	2 (2.4)	NS	NS	NS	NS	NS
**Overall etiology results**												
Bacterial etiologies incl. AFB (% = n*100/b)	520 (29.0)	355 (39.0)	184 (65.0)	171 (27.2)	0 (0.0)	132 (30.1)	33 (11.2)	**<0.001**	**<0.001**	**0.002**	**<0.001**	**<0.001**
Viral detection (% = n*100/a)	298 (16.1)	135 (14.4)	21 (7.5)	114 (17.4)	13 (7.9)	68 (15.4)	82 (26.1)	NS	**0.033**	NS	**<0.001**	NS
Identified etiologies (% = n*100/c)	749 (42.9)	449 (50.6)	192 (69.8)	257 (42.0)	13 (8.8)	178 (41.9)	109 (37.8)	**<0.001**	**<0.001**	**0.004**	**<0.001**	**<0.001**

IQR: interquartile range; NS, not significant (NS is displayed when p value is >10%); PCR, polymerase chain reaction;

ALRI = acute lower respiratory infection; Other viruses include Parainfluenza viruses, Bocaviruses, Adenoviruses.

**Hyperleukocytosis: Leukocytes count ≥11 for 5–10 year-olds or ≥10 for ≥11 year-olds.

**Elevated neutrophils count ≥6 for 5–10 year-olds or ≥7.5 for ≥11 year-olds.

*** % = proportion of AFB-positive by # of patients tested for AFB.

‡ % = proportion of positive results by # of patients tested.

† % = proportion of positive bacteria by # of patients tested positive by sputum or blood culture.

^1^ and ^2^ % = proportion of positive bacteria by # of patients tested positive by sputum and blood culture respectively.

^ % = proportion of virus of interest by # of positive PCR.

### Microbiological etiology

**Figure 2:**
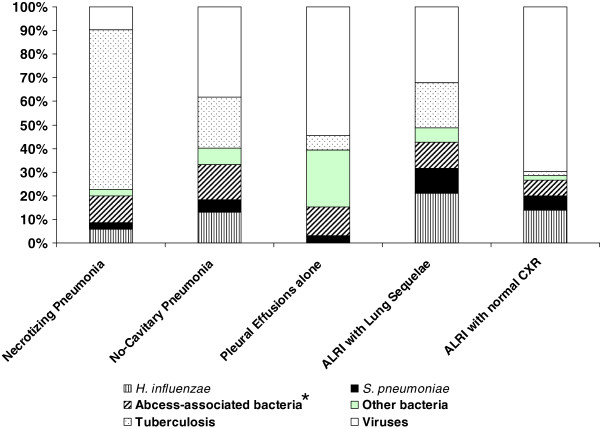
Etiology of community-acquired acute lower respiratory infection in ≥5 year-old patients hospitalized during April 2007 – December 2009, Kampong Cham and Takeo provinces, Cambodia (749 patients with positive findings). *Abcess-associated bacteria include *K. pneumoniae*, *B. pseudomallei*, *P. aeruginosa*, *S. aureus*.

While nasopharyngeal/throat swabs (97.4%) and blood samples (89.0%) could be easily obtained for each patient, sputum samples were only able to be collected in 1,053 patients (12.8% and 54.8% in 5–14 year-olds and >15 year-olds respectively). Sputum was only available for culture in 888 (46.6%) patients ranging from 21.9% among cases presenting with pleural effusions alone to 64.0% among pneumonia cases. In contrast, sputum specimens were more readily available for direct AFB examination. This major gap was mainly explained by incorrect sputum specimen collection that resulted in salivary contamination (monthly mean rate 13.2%, range: 5.3% - 23.9%). Overall, a combination of these tests identified bacterial and viral etiology in 749 (42.9%) patients ranging from 8.8% positive results in patients with pleural effusions alone to 69.8% in patients with necrotizing pneumonia (Table 1). Mixed infections were not associated with severity (p = 0.192) or a specific type of ALRI, except between the groups of necrotizing pneumonia and non-cavitary pneumonia (7.3% versus 1.6%, p < 0.001) (Table 1). Of the identified pathogens, bacteria (73.9%) were the most frequent etiology in pneumonia with predominantly AFB (41.2%; 67.7% in necrotizing pneumonia and 21.5% in non-cavitary pneumonia), followed by *Haemophilus influenzae* (9.8%) and *Streptococcus pneumoniae* (4.5%) (Figure 2). Bacteria also predominated in lung sequelae-associated ALRI (67.9%) consisting of *H. influenzae* (21.1%), AFB (19.3%), Gram-negative bacteria (11.0%) and *S. pneumoniae* (10.6%). In contrast, a viral etiology was predominant in ALRI patients with a normal CXR (67.5%) including the pleural effusions group (54.5%) and ALRI with normal CXR group (74.3%) (Figure 1).

Of the 300 (16.8%) positive sputum or blood cultures, abscess-prone Gram-negative bacteria (39.6%) (*i.e. Klebsiella pneumoniae, Burkholderia pseudomallei, Pseudomonas aeruginosa*) and *H. influenzae* (38.0%) were most frequent, followed by *S. pneumoniae* (17.7%). Of the 305 identified viruses, the three most common viruses included rhinoviruses (49.5%), respiratory syncytial virus (17.7%), influenza viruses (12.1%) and coronaviruses (10.2%), regardless of the diagnostic groups (Table 1).

### Mortality and severity

During hospitalization, 61 (3.2%) patients died, of which the following etiologic agents were identified in 22 (36.1%) patients including 7 (31.8%) with *Burkholderia pseudomallei*, 3 with AFB, 3 with various bacteria, 7 with viruses (including 5 rhinoviruses alone) and 2 with mixed infections. The median age of the deceased patients was 50 years (range: 6 – 87). When compared to patients who improved at discharge, those with who died in hospital were more likely to have *B. pseudomallei* infection (4.1% vs. 31.8%, p < 0.001), diagnosis of pneumonia (45.3% vs. 70.5%, p < 0.001), and elevated blood neutrophils count (34.7% vs. 49.2%, p = 0.024); no differences were observed in gender, age, with co-morbid conditions, and in combination with other pathogens.

A total of 188 (9.8%) patients met the criteria for severe cases, of which the most frequent signs consisted of tachycardia (77.7%), tachypnea (93.1%) and low oxygen saturation (39.4%). Pneumonia with (8.0%) and without (56.4%) cavitations and ALRI with normal CXR (22.3%) accounted for the majority of the severe cases. When compared with non-severe cases, severe cases tended to be younger (median age 25.5 years vs. 55 years, P < 0.001); 42.6% of the severe cases were aged 5–15 years and the median age of severe cases in the non-cavitary pneumonia group was 8 years (Table 1). Other univariate analyses of differences between severe and non-severe cases are illustrated in Table 2. Multivariate analyses revealed the following variables independently associated with severity: <50 years of age (69.1% vs. 39.8%; adjusted OR (aOR) 2.9, p < 0.001), elevated blood neutrophil count (56.4% vs. 32.9%; aOR 2.0, p = 0.001), non-cavitary pneumonia (56.4% vs. 32.9%; aOR 3.6, p < 0.001), mortality (9.0% vs. 2.6%; aOR 3.2, p = 0.002), shorter (<5 days) time to admission (62.8%, aOR 2.0, p = 0.005) and wheezing (33.5% vs. 11.5%, aOR 2.5, p < 0.001) (Table 3).

**Table 2:** Univariate analyses: factors associated with severity among ≥5 year-old patients hospitalized with community-acquired acute lower respiratory infection, April 2007 - December 2009, Kampong Cham and Takeo provinces, Cambodia (N = 1,904)

	**Severe cases**		**Non-severe cases**		**p values**
Total numbers (N = 1904)	**188**	9.9%	**1716**	90.1%	
**Median age in years (range)**	25.5	8-56	55	41-66	**<0.001**
**Mean age in years**	32.1		51.8		**<0.001**
**Age groups in years**					
5-14	80	**42.6%**	74	4.3%	**<0.001**
15 - 49	50	26.6%	609	35.5%	**0.019**
≥50	58	30.9%	1033	60.2%	**<0.001**
**Gender, male**	106	56.4%	835	48.7%	0.068
**Signs and symptoms**					
Wheezing	63	33.5%	198	11.5%	**<0.001**
Dry cough	84	44.7%	435	25.3%	**<0.001**
**Time interval between presentation and symptoms onset in days**					
Median (IQR)	4	2-7	6	4-9	**<0.001**
Mean	4.9		6.4		**<0.001**
**Duration of hospital stay in days**					
Median (IQR)	5	3-7	7	4-11	**<0.001**
Mean	6.1		8.6		**<0.001**
**Reports of medicine use prior to admission**					
no antibiotics used	97	51.6%	835	48.7%	NS
Unidentified drugs used	62	33.0%	714	41.6%	**0.028**
Antibiotics used	29	15.4%	154	9.0%	**0.007**
**Patient outcomes**					
Deceased	17	**9.0%**	44	2.6%	**<0.001**
Cured or improved	132	70.2%	1132	66.0%	NS
Transferred	32	17.0%	450	26.2%	**0.008**
Escaped	7	3.7%	90	5.2%	NS
**Co-morbidities**					
Cardiovascular disorder	28	14.9%	190	11.1%	NS
Diabetes mellitus	20	10.6%	175	10.2%	NS
Renal disorder	18	9.6%	113	6.6%	NS
Liver disorder	0	0.0%	16	0.9%	NS
**Laboratory results**					
Leukocytosis	106	57.3%	552	32.3%	**<0.001**
Neutrocytosis	106	**58.6%**	523	34.0%	**<0.001**
Lymphocytosis	14	8.5%	85	5.6%	NS
**Patients tested by microbiology method**					
Blood culture	168	89.4%	1527	89.0%	NS
Sputum culture	47	25.0%	843	49.1%	**0.002**
Blood or sputum culture	173	92.0%	1616	94.2%	NS
Sputum smear for acid-fast bacilli (AFB)	113	60.1%	1569	91.4%	**<0.001**
Nasopharyngeal swabs	184	97.9%	1671	97.4%	NS
Samples collected for bacterial & AFB etiologies	173	92.0%	1623	94.6%	NS
Samples collected for bacterial & viral etiologies	169	89.9%	1579	92.0%	NS
**Bacterial or viral yield**					
Positive blood culture	9	5.4%	24	1.6%	**0.002**
Positive sputum culture	15	31.9%	257	30.5%	NS
Positive blood or sputum culture	24	13.9%	276	17.1%	NS
Positive smear for AFB	13	11.5%	251	16.0%	**<0.001**
Positive PCR for viruses	55	29.9%	250	15.0%	**<0.001**
**Bacterial etiologies**					
*H. influenzae*	5	2.9%	109	6.7%	0.074
*S. pneumoniae*	3	1.7%	50	3.1%	NS
*B pseudomallei*	5	2.9%	23	1.4%	NS
*Klebsiella pneumoniae*	5	2.9%	38	2.4%	NS
*P. aeroginosa*	1	0.6%	36	2.2%	NS
*S. aureus*	1	0.6%	10	0.6%	NS
AFB	13	7.5%	251	15.5%	**0.030**
**Viral etiologies**					
Rhinovirus	32	17.4%	119	7.1%	**<0.001**
Respiratory syncytial virus	6	3.3%	48	2.9%	NS
Influenza viruses	4	2.2%	33	2.0%	NS
Coronaviruses	6	3.3%	25	1.5%	NS
Human metapneumovirus	4	2.2%	10	0.6%	0.054
Other viruses	5	2.7%	21	1.3%	NS
**Mixed infections (% = # of the positive results)**					
>1 pathogen (AFB, bacterium or virus)	3	3.5%	60	8.9%	NS
>1 bacterium identified	1	1.2%	25	3.7%	NS
AFB and ≥1 bacterium identified	1	1.2%	29	4.3%	NS
Co-infections AFB & virus	0	0.0%	26	3.9%	NS
Co-infections virus-virus	2	2.3%	6	0.9%	NS
**Overall etiology results**					
Bacterial etiologies (AFB or other bacteria)	36	20.8%	498	30.7%	**<0.001**
Viral and bacterial etiologies	86	50.9%	674	42.7%	**<0.001**

**Table 3:** Independent risk factors for severe community-acquired acute lower respiratory infection among hospitalized ≥5 year-old patients, from April 2007 to December 2009, Kampong Cham and Takeo provinces, Cambodia

	**Severe cases**		**Non-severe cases**			
	**N = 188**	**%**	**N = 1716**	**%**	Adjusted OR*	p value**
Age groups						
5 - 49 years	130	69.1%	683	39.8%	2.9	**<0.001**
>50 years	58	30.9%	1033	60.2%	reference	
Pneumonia						
No cavities	106	56.4%	564	32.9%	reference	
With cavities	15	8.0%	274	16.0%	0.33	0.029
Pleurisy alone	11	5.9%	158	9.2%	0.51	**0.133**
Sequelae prior infection	14	7.4%	441	25.7%	0.21	**<0.001**
ALRI with normal CXR	42	22.3%	279	16.3%	**0.52**	**0.018**
Neutrophils						
Normal	75	39.9%	1063	61.9%	reference	
Elevated	106	56.4%	523	30.5%	**2.03**	**0.001**
Outcome						
Improved	132	70.2%	1132	66.0%	reference	
Deceased	17	9.0%	44	2.6%	**3.20**	0.002
Wheezing						
No	125	66.5%	1494	87.1%	**reference**	
Yes	63	33.5%	198	11.5%	**2.50**	**<0.001**
Time to admission						
<5 days	118	62.8%	653	38.1%	**2.00**	
≥5 days	65	34.6%	984	57.3%	**reference**	**0.005**

*Adjusted by viral etiology, bacterial etiology, antibiotics intake prior admission, time to admission and gender.

**By Wald test.

Overall, 261 (13.7%) patients presented with wheezing on admission of which 63 (24.1%) were classified as severe cases. Among the 5–14 year-olds, 63.4% had wheezing compared with 11.1% in the >50 year age group. The frequency of wheezing was significantly higher in acute bronchitis (32.8%) compared with other diagnostic groups. Other independent variables that were observed with wheezing included having an elevated-neutrophil-count (19.4% vs. 10.7%; aOR 1.8, p = 0.001), being diagnosed with a rhinovirus infection (32.7% vs. 12.4%; aOR 2.7, p < 0.001) and being classified as a severe case (33.5% vs. 11.7%; aOR 2.3, p < 0.001). Notably, asthma as a co-morbid condition was insufficiently recorded in the CRF; only 17 (0.9%) asthmatic patients were reported and 1,510 (79.3%) CRF had missing values.

### Factors associated with identification of microorganisms

Age was significantly associated with microbiological yield. The proportion of patients with identifiable viruses among those presenting with pneumonia (Chi-squared test for trend 76.3, p < 0.001) or those with ALRI and normal CXR (Chi-squared test for trend 16.5, p < 0.001) decreased with increasing age while proportions of patients with identified bacteria were higher among the ≥10 year-olds compared with that of the <10 year-olds (17.6% versus 4.3% respectively, p = 0.002). Most patients (51.1%) reported use of antibiotics (10.3%) or unknown drugs (40.8%) prior to hospital admission. No associations were observed between pre-admission antibiotic use and negative sputum (p = 0.854) or blood (p = 0.275) culture. When considering the influence of other factors for virus identification in a multivariate analysis, wheezing (31.9% vs. 13.8%, aOR 2.2, p < 0.001), 5–14 year-old children (54.6% vs. 14.0%; aOR 1.6, p = 0.001) and short (<5 days) time to admission (22.6% vs. 11.5%; aOR 1.9, p < 0.001) had a positive association with viral yield. Of all detected viruses, only rhinovirus was associated with wheezing (32.7% vs. 12.3%, aOR 2.7, p < 0001), adjusting for age.

Interestingly, of the 796 patients whose blood and sputum specimens were negative for bacteria, 121 (15.2%) patients presented with an elevated blood lymphocyte count or normal neutrophil count on admission; these 121 patients had no radiographic images indicative of TB, no necrotizing pneumonia, and no use of antibiotics preceding hospitalization. The median age was 55 years (range 6 – 85) and 50.4% were males. Viral etiology was assigned to 28 (21.6%) patients including rhinoviruses (35.7%), RSV (42.9%), influenza viruses (14.3%) and parainfluenza viruses (7.1%). Of these, 9 were diagnosed as having pneumonia (i.e. 4 rhinoviruses, 3 influenza viruses and 2 RSV), 10 had lung sequelae-associated ALRI (i.e. 5 RSV, 3 rhinoviruses, 1 influenza virus, 1 parainfluenza virus), 2 had pleural effusions alone (i.e. 2 RSV) and 7 had acute bronchitis (i.e. 1 parainfluenza virus, 3 RSV and 3 rhinoviruses). Of the 5 (3.7%) reported deceased, only one virus (RSV) was identified in a 53 year-old male presenting with severe non-cavitary pneumonia and no reported co-morbid conditions.

## Discussion

This study is notable in many ways. To our knowledge, it is the first report of a comprehensive picture of radiographically confirmed pneumonia and other ALRI that resulted in hospitalization in a low-income tropical country of Southeast Asia. In addition, only a few studies have been conducted in semi-rural hospitals in a population of all ages using a wide range of diagnostic tools to identify viruses and bacteria causing ALRI [5-7,15,16].

The primary finding relates to categorizing various forms of ALRI based on radiographic patterns combined with the identification of pathogens. Indeed, bacterial etiologies varied significantly from one form of ALRI to another. Pneumonia, which is mainly caused by bacteria in hospitalized persons aged ≥5 years, accounted for most of the ALRI cases. However, a large proportion of the ALRI patients had radiographic imaging of lung sequelae, most probably caused by tuberculosis or undertreated pneumonia.

Depending on the immune response of the host, TB generally evolves slowly (3–8 weeks) before the onset of symptoms [17,18]. However, in this study, we were surprised to have found AFB to be the most common pathogen in hospitalized patients with acute pneumonia. Although Cambodia is known as one of the countries with the highest TB prevalence and incidence reported in the world (http://www.who.int/tb/country/en/index.html), the interpretation of these results is unclear. It is possible that the history of illness was poorly recalled by the patient or a mixed infection existed with a non-TB respiratory pathogen which may have led to hospitalization. On the other hand, there is emerging evidence across the world and particularly shown in African studies, that *M. tuberculosis* is commonly present in acute community-acquired pneumonia in children [15,19] and adults [20], particularly in settings of high TB prevalence. One suggested explanation is the increased susceptibility to bacterial infection (particularly pneumococcal infection) by prior *M. tuberculosis* infection as demonstrated by experimental studies [21]. In the present study, 21% of tuberculosis cases were co-infected with other bacteria, of which *S. pneumoniae* was the main (43%) pathogen identified. Our findings imply that expanding the TB screening criteria to include acute pneumonia might be considered. Of note, Cambodia is planning to develop a multi-symptoms approach to increase cost-effective routine screening for TB.

Our second main finding also indicates that a non-negligible proportion of patients presenting with cavitary lung imaging suggestive of TB were affected by other abscess-associated bacteria (e.g. *K. pneumoniae, B. pseudomallei, S. aureus*, etc.…). Prior to this study, these bacteria tended not to be recognized by local clinicians who empirically treated patients with a penicillin A-based therapy, an antibiotic that is not effective on some naturally resistant strains of Gram-negative bacteria such as *B. pseudomallei*[22,23]. In adult patients with non-cavitary pneumonia, common pathogens causing community-acquired pneumonia in West and Far-East Asia include *S. pneumoniae, Mycoplasma pneumoniae, Chlamydia pneumoniae* and *Legionella pneumoniae*[15,16,24-26]. First, atypical pathogens (i.e. *C. pneumoniae* and *M. pneumoniae*) were rarely detected in our hospitalized patients: of a subgroup of sputum specimens from a group of 304 randomly selected study patients, only 3 tested positive by PCR: 2 for *M. pneumoniae* and 1 for *C. pneumoniae* (IPC, unpublished data). This result was also observed elsewhere [20,27,28] and was consistent with another recent study of community-acquired pneumonia among immuno-compromised adults in Cambodia [27]. Secondly, we found a lower frequency of *S. pneumoniae* compared with other bacteria*.* Because *S. pneumoniae* is known to be fragile, some related infections might have gone undetected as cultures were performed at IPC after a certain delay due to transportation of specimens [29]. The extent of undetected *S. pneumoniae* infection may be suggested by the following sub-study: of a random sample of 24 ALRI cases (median 58 years of age, range 18 – 78) for which we also collected urine samples, three (13%, 95% confidence interval 3% - 32%) patients tested positive for *S. pneumoniae* antigen by Binax NOW® rapid immunochromatographic assay (Scarborough, ME). (IPC, unpublished data). Accounting for this underestimation, one might expect *S. pneumoniae* or *H. influenzae* –related respiratory infections to reach ~60% of the bacterial etiology of acute pneumonia. As a Global Alliance Vaccine Initiative or GAVI - eligible country, Cambodia introduced *H. influenzae b* vaccines in 2010 and will probably do so in the near future for pneumococcal conjugate vaccine.

RSV and influenza viruses have been recognized as the leading causes of severe ALRI in infants and young children [30,31]. These viruses can also cause severe ALRI in certain at-risk groups. In our study, viral pathogens, especially rhinoviruses and RSV were not only limited to these high risk groups but were common in older children. Moreover, age was determined as a major confounding factor when identifying risks associated with viral etiologies. Only time-to-admission was independently associated with the viral identification rate, a finding that suggests that viral infections were under-detected in many bacterial ALRI patients who were admitted late in the course of illness [32].

The extent to which these viruses cause direct and severe ALRI is difficult to assess in our study, particularly in absence of a control group. Indeed, certain viruses were demonstrated to be associated with severe pneumonia [33]; however, detection of some respiratory viruses, especially rhinoviruses, was frequently found in non-ill or non-respiratory infected control groups when using highly sensitive molecular diagnostic techniques [13,33-39]. Interestingly we identified viruses in ALRI patients with little evidence of associated bacterial infection. Further virologic analyses would be worthwhile to determine the factors comparing pneumonia cases with no evidence of bacterial infection to those associated with superinfection.

Symptoms associated with severity as defined by the parameters of the study were highly prevalent in children; the reasons are unclear or potentially explained by several factors such as having acute pneumonia and/or underlying asthma. Interestingly, wheezing was also independently associated with viral respiratory infection. This relationship between viral infection, asthma (wheezing as its surrogate), and disease severity have been observed more recently. Indeed, recent studies suggest that respiratory infections caused by rhinoviruses or RSV in infancy can lead to the development of asthma at an older age [40,41], which could be exacerbated by viral infections [34,42,43]. Recognizing the high frequency of wheezing in children with pneumonia (~62%) and its association with hyperleucocytosis in our study, it is possible that asthma could either directly cause a severe case or lead indirectly to signs of severity via superinfection and subsequent pneumonia. Education programs designed to recognize asthmatic symptoms, teach management strategies, and encourage the prevention of asthma should be urgently implemented.

These preceding findings need to be interpreted in light of some major limitations. First, the rate of bacteria identification in this study was low compared to other studies using routine clinical diagnostic techniques [15,27,44]. Approximately 17% of bacterial diagnoses were achieved through the use of blood or sputum cultures. This relatively low yield contrasted with a much higher proportion of pneumonia-related patients (~40%) who had an elevated blood neutrophil count, a parameter that is indicative of bacterial infection. Various factors contributed to this low yield, including the probable high intake of antibiotics, which can be bought without a prescription and frequently are administered irrespective of the proper dosage and length in Cambodia [45]. Since there was only a one-time collection of sputum and blood for each patient, a high bacterial yield was not expected. Moreover, sputum tests, which are commonly used to diagnose pneumonia, may lack specificity without evocative clinical information, have poor sensitivity or are frequently contaminated by saliva or upper respiratory tract colonization. This resulted in having to discard numerous sputum specimens prior to culture in our study. The use of invasive procedures such as brochioalveolar lavage or sputum induction was difficult to introduce, particularly in children. Second, the burden of TB in this study was probably largely underestimated as cultures for *M. tuberculosis* were not performed [46].

## Conclusions

Overall, introducing a bacteriological laboratory capacity for ALRI surveillance has proven to be a major investment in settings of limited resources and experiences, requiring sustained training and monitoring to ensure reliability and consistency. Despite these challenges, bacteriological testing provides useful insights and contributions to virologic surveillance [47] and above all, directly benefits patients' care and treatment.

## Competing interest

All authors declared no competing interest.

## Authors' contributions

Conceived and designed the study: SV, PB, BG and CM. Performed the study: BR, LB, PC, SL, SG. Clinical data collected: VT, PLT, BR. Analyzed the data: SV, SG, SL. Contributed reagents/materials/analysis tools: BG, SH, SR, PB. Wrote the paper: SV, PB. Critical review of the paper: All. Found funding: SV, PB. All authors read and approved the final manuscript.

## Pre-publication history

The pre-publication history for this paper can be accessed here:

http://www.biomedcentral.com/1471-2334/13/97/prepub
